# The Impact of Face Inversion on Animacy Categorization

**DOI:** 10.1177/2041669517723653

**Published:** 2017-08-11

**Authors:** Benjamin Balas, Amanda E. van Lamsweerde, Amanda Auen, Alyson Saville

**Affiliations:** Department of Psychology, North Dakota State University, Fargo, ND, USA

**Keywords:** face recognition, expertise, animacy, ERPs, categorical perception

## Abstract

Face animacy perception is categorical: Gradual changes in the real/artificial appearance of a face lead to nonlinear behavioral responses. Neural markers of face processing are also sensitive to face animacy, further suggesting that these are meaningful perceptual categories. Artificial faces also appear to be an “out-group” relative to real faces such that behavioral markers of expert-level processing are less evident with artificial faces than real ones. In the current study, we examined how categorical processing of real versus doll faces was impacted by the face inversion effect, which is one of the most robust markers of expert face processing. We examined how explicit categorization of faces drawn from a real/doll morph continuum was affected by face inversion (Experiment 1) and also how the response properties of the N170 were impacted by face animacy and inversion. We found that inversion does not change the position or steepness of the category boundary measured behaviorally. Further, neural markers of face processing are equally impacted by inversion regardless of whether they are elicited by real faces or doll faces. On balance, our results indicate that inversion has a limited impact on the categorical perception of face animacy.

## Introduction

The perception of face animacy, which is the distinction between real and artificial faces, appears to be categorical. That is, multiple studies suggest that there is a meaningful perceptual boundary between real and artificial faces. For example, when participants are asked to categorize faces that fall along a morph continuum spanning real and doll appearance as either real or artificial, those judgments exhibit the sigmoidal shape that typically reflects categorical perception (Looser & Wheatley, 2010). This effect has been observed with both doll faces and computer-generated (CG) faces (Balas & Horski, 2012; Balas & Tonsager, 2014), suggesting that the particular type of artificial face does not matter very much—artificial appearance is categorically distinct from real face appearance in both cases. In addition to these behavioral demonstrations that participants make nonlinear responses regarding animacy across an ostensibly continuous spectrum of real and artificial face appearance, neural indices of face categorization also suggest that there is a meaningful boundary between these types of face stimuli that interacts with other face categories. Face aftereffects resulting from prolonged viewing of real or artificial stimuli do not transfer across species boundaries, for example (Koldewyn, Hanus, & Balas, 2014), suggesting that responses to real and artificial human faces don’t reflect boarder cognitive processes, but instead reflect perceptual processing. Similarly, face animacy impacts the differential processing of face species such that sensitivity to own versus other-species appearance is reduced in artificial faces (Balas & Koldewyn, 2013). Besides these instances of face animacy impacting other categorical face properties, there is also evidence that multiple indices of face-specific processing in the ventral visual stream are sensitive to real versus artificial appearance (Looser, Guntupalli, & Wheatley, 2013; Wheatley, Weinberg, Looser, Moran, & Hajcak, 2011), further suggesting that these stimuli differ categorically at later stages of cortical processing.

These categorical differences between real faces and various kinds of artificial faces (e.g., dolls or CG faces) also appear to signal that artificial faces are in some ways less face-like than real ones. While dolls and CG faces are easily perceived as faces and have most of the same basic face geometry as real people, they are discriminable from real faces (Balas & Tonsager, 2014; Farid & Bravo, 2011) and nonetheless may be something of an out-group, based on observers’ comparative lack of exposure to members of these categories. For example, like other-race faces (Meissner & Brigham, 2001) and other-age faces (Kuefner, Macchi Cassia, Picozzi, & Bricolo, 2008; Rhodes & Anastasi, 2012), artificial faces are more poorly remembered than real faces, even when identity (and by extension, other variables) are well-matched across real and CG stimuli (Balas & Pacella, 2015, submitted). The well-known other-race effect is also diminished in synthetic faces relative to real faces (Crookes et al., 2015), further suggesting that artificial faces are not necessarily processed by the same expert-level mechanisms that are applied to real faces. These categorical differences between real and artificial faces may have important consequences for a wide range of studies that have made use of various types of artificial faces but also presents several interesting questions regarding how animacy is perceived in face images, such as (a) how does the boundary between real and artificial faces determine the processes applied to different images and (b) how is the boundary between real and artificial faces *determined by* manipulations that are known to affect expert face processing?

With regard to the latter question, the face inversion effect (or FIE; Yin, 1969) is an interesting feature of face processing that we suggest is important to consider in the context of real versus artificial face recognition. Briefly, the FIE refers to several related phenomena that all reflect disproportionately poor processing of upside down faces compared with upright faces (see Valentine, 1998 for a review). Over several decades, the inversion effect has been used variously as a tool for measuring face expertise, as an index of “configural” or “holistic” processing (Diamond & Carey, 1986), or simply as a useful way to control for many low-level properties of face images.

Determining how face inversion interacts with processing face animacy is interesting to consider for several reasons. First, with regard to the potential for artificial faces to be an out-group relative to real faces, this could imply that the processes applied to real faces (which we assume entail the FIE) are not applied to artificial faces, which would reduce the FIE for these stimuli. Second, the use of inverted faces as a control for low-level properties is potentially very important to consider when evaluating the results of prior studies that have relied upon ratings or recognition/discrimination behavior across a continuum of faces spanning real and artificial face appearance. The critical result that such behavioral data exhibit nonlinearity across a putatively linear continuum relies heavily on the assumption that the underlying continuum is actually uniform. That is, though the faces used in such tasks are typically labeled according to some uniform step-size along the relevant spectrum (e.g., 10% morph levels), there is no guarantee that those steps are actually the same size perceptually across that range. Face inversion thus provides a useful means of establishing whether the steepness of that boundary truly reflects a face-specific process, or whether instead it may reflect some lower level feature of the stimuli. Finally, the impact of face inversion on the responses made to real and artificial faces is itself an important basic question that should reveal what perceptual properties are necessary to distinguish between these two classes of stimuli and how face-specific mechanisms differ from more general aspects of object processing in terms of the response to face animacy.

Our goal in the present study was therefore to determine the impact of face inversion on real versus artificial face categorization. In two experiments, we examined behavioral (Experiment 1) and neural (Experiment 2) responses to real faces and doll faces in the context of a category judgment to examine how inversion impacted both types of faces. In our first task, participants were asked to categorize upright and inverted face according to animacy across a continuum spanning real and doll appearance. This task allowed us to estimate the parameters of the psychometric curve obtained from the categorization data to determine if the position or steepness of the category boundary between real and doll faces changed as a function of orientation. In our second task, we recorded electroencephalogram (EEG) responses to real and doll faces presented upright and inverted to determine how the N170 component (a widely studied face-sensitive event-related potential [ERP] component—Rossion & Jacques, 2008—that is also sensitive to face inversion—Eimer, 2000; Rossion et al., 2000) was affected by face category and orientation. In this case, the response properties of the N170, specifically its amplitude and latency, allowed us to determine how neural responses to real and doll faces might be impacted by inversion. In both cases, we predicted that face inversion might weaken the category boundary between real and artificial faces such that the distinction between real and doll appearance would be less clear when faces were presented upside down. Instead, we found little evidence that inversion affected the categorical processing of real versus doll faces, suggesting that the perceptual cues supporting the distinction between real and artificial faces are robust to this manipulation. We discuss this result in the context of other related results on artificial face perception and speculate on what our result implies about the nature of categorical processing of face animacy at early versus late stages of visual processing.

## Experiment 1

In our first experiment, we asked how face orientation (upright or inverted) affected the categorization of faces according to animacy (real or doll) across a morph continuum spanning these end points. To do so, we asked naïve observers to complete a real/doll categorization task subject to brief presentation times, recording both the accuracy and latency of their responses.

### Methods

#### Participants

We recruited 26 adult observers (13 female) from the NDSU Undergraduate Psychology Study Pool. Participants ranged in age from 18 to 23 years old and self-reported normal or corrected-to-normal vision. Prior to beginning the experiment, all participants provided written informed consent following procedures established by the NDSU IRB and consistent with the principles articulated in the Declaration of Helsinki.

#### Stimuli

**Figure 1.**
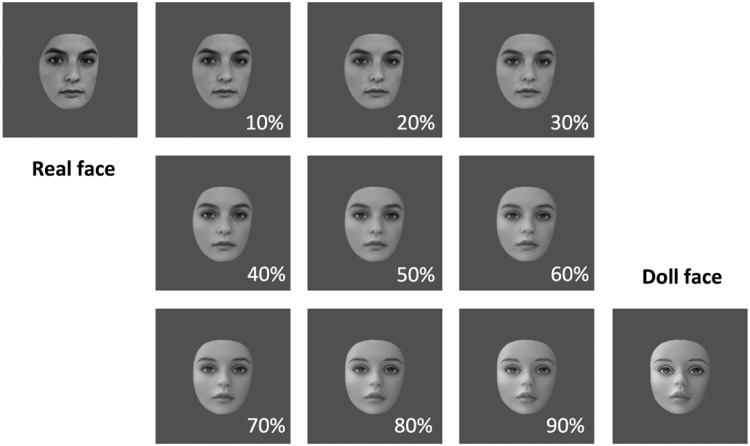
An example of a real/doll morph continuum used in Experiment 1. In Experiment 2, only the end points of this spectrum were used.

We created four unique continua of faces spanning real and artificial appearance by morphing together images of human faces with images of dolls. The original images of both humans and dolls were 256 × 256 pixels in size and rendered in grayscale. Each image depicted a unique individual with the hairline cropped out, but the jawline left intact (Figure 1). Morphing operations were carried out using Norkross Morph for the MacOS by identifying multiple fiducial points on both faces that could be placed in correspondence before warping one face (the human face) into the other (the doll face). We divided each morph continuum into 11 steps by exporting images along each continuum in 10% steps. The entire stimulus set was thus comprised of 44 images, all of which were matched for mean pixel intensity.

#### Procedure

Participants in this experiment completed a real/doll categorization judgment subject to rapid presentation of our stimulus images. On each trial, participants were presented with a single image from one of the four morph continua, which could be depicted either in the upright orientation or upside down (inverted). Each image was presented for 200 ms, followed by a grayscale noise mask depicting 1/f fractal noise that we presented for 500 ms. Trial order was pseudorandomized for each participant, and each stimulus was repeated 6 times in each orientation, for a grand total of 528 trials.

Participants completed the task seated at a comfortable viewing distance from a 1,200 × 800 LCD monitor. At this viewing distance, the stimuli subtended approximately 3 to 4 degrees of visual angle, though this varied somewhat across participants. All stimulus timing parameters and response collection routines were implemented via custom scripts written using the Psychtoolbox v3.0 extensions for MATLAB (Brainard, 1997; Kleiner, Brainard, & Pelli, 2007; Pelli, 1997).

### Results

#### Accuracy

**Figure 2.**
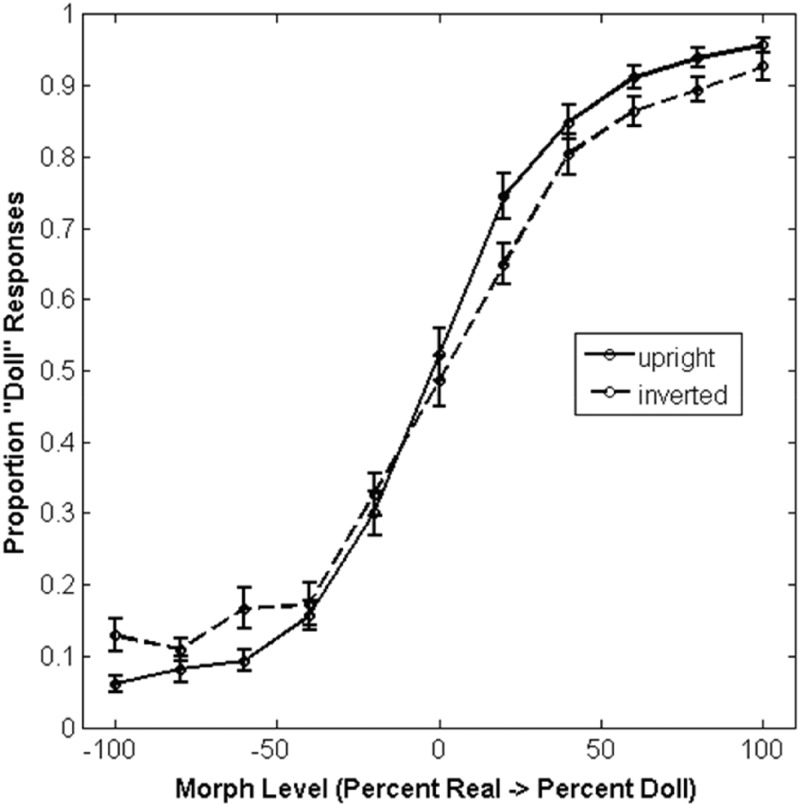
The mean proportion of trials at each morph level categorized as doll faces. Error bars depict ±1 standard error of the mean.

To analyze the data collected in Experiment 1, we fit a cumulative normal function to each participant’s responses. Specifically, for each morph level in our stimulus continuum, we calculated the proportion of “Doll” responses made to face images at that level and identified values of the free parameters (α, β, γ, and λ) that minimized the error between the raw data and the psychometric curve (see Figure 2 for average responses for upright and inverted faces across all morph levels). Briefly, these free parameters reflect distinct properties of the curve’s shape: Alpha (α) values reflect the position of the point of subjective equality, beta (β) reflects the steepness of the psychometric curve in the linear region, and both gamma (γ) and lambda (λ) reflect the position of the horizontal asymptotes relative to floor and ceiling performance. We carried out curve-fitting procedures using MATLAB’s “cftool” interface and obtained robust fits for 24 of our 26 participants (mean *R*^2^ values ∼.94). The remaining two participants were excluded from this analysis because examination of their data suggested that they failed to follow task instructions adequately, leading to poor performance across all morph levels that likely reflected inattentiveness to the stimuli.

Having obtained values of all four free parameters of the psychometric function for each participant, we first observed that neither alpha, *t*(23) = 1.64, *p* = .12, nor beta values, *t*(23) = 1.63, *p* = .12, were significantly affected by inversion, suggesting that responses in the putative boundary region between real and doll faces did not change when faces were turned upside down. We also note that while previous studies have found that the midpoint of psychometric curves reflecting real/artificial face categorization responses was significantly shifted toward the “Real” end of the spectrum, we did not replicate this feature of the data in the current study. However, the lack of such a shift away from the physical midpoint of the morph continuum does not by itself explain our lack of an inversion effect in the alpha/beta parameters reported earlier. Unlike matching or discrimination tasks that have an objective “floor” for chance performance, participants in our task could have responded with either category at any point along the spectrum. Thus, even though the midpoint of our psychometric curve for upright faces is not shifted away from the midpoint of the spectrum spanning real and artificial appearance, inversion could still have exerted a systematic effect on appearance such that participants might have labeled inverted faces as artificial with increased frequency.

We continued by examining the effect of inversion on gamma (γ) and lambda (λ). Both of these parameters reflect participant’s asymptotic accuracy along our morph continuum: Gamma reflects a “guess rate” that is affected by errors at the “Real” end of the continuum in our task, while lambda reflects a “lapse rate” that is affected by errors at the “Doll” end of the spectrum. In both cases, we had a clear directional hypothesis for each parameter. If inversion affects real and artificial faces similarly, gamma and lambda should be larger when inverted faces were presented to the participant, due to increased errors at both ends of the continuum. We examined each outcome using single-sample, one-tailed *t* tests comparing the observed mean to zero.

We observed a significant effect of face inversion on gamma values, *t*(23) = 2.28, 95% confidence interval (CI) of the difference [0.0036, 0.076], Cohen’s *d* = 0.46, *p* = .016, suggesting that inversion does affect the categorization of real faces. With regard to lambda values, we found that this effect did not reach significance, *t*(23) = 0.83, 95% CI of the difference [−0.027, 0.063], Cohen’s *d* = 0.17, *p* = .21. This suggests that inversion does not systematically affect the categorization of artificial faces.

#### Response latency

**Figure 3.**
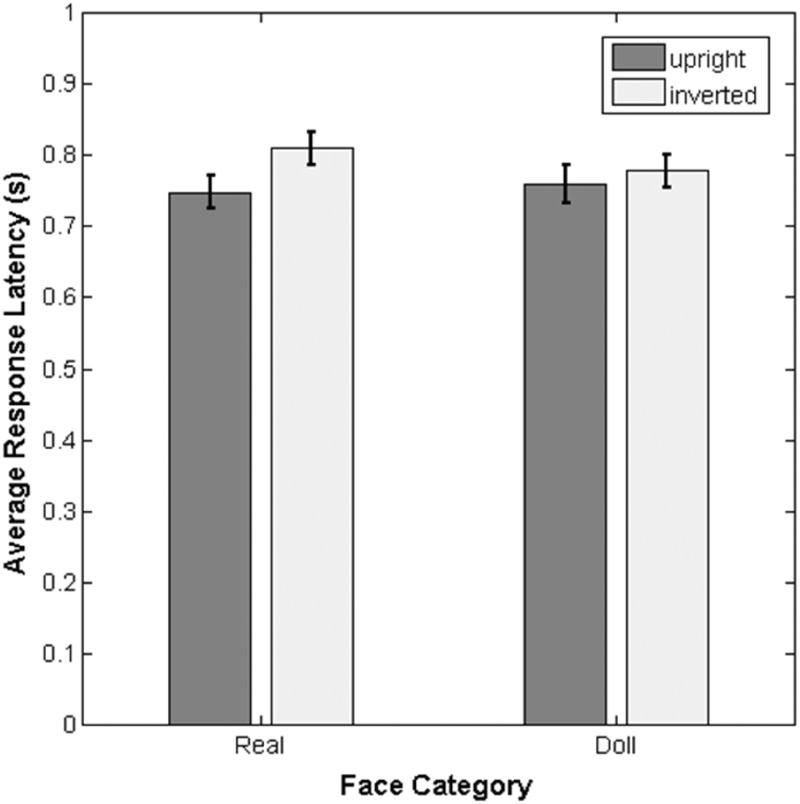
The mean response latency across participants for correct responses to real and doll faces. Error bars depict ±1 standard error of the mean.

To complement our analysis of labeling performance, we also examined the response latency for correct responses at both the “Real” and “Doll” end of the morph continuum. Unlike our previous analysis, we did not perform any curve-fitting prior to carrying out statistical tests. There are two reasons for this: First, neither the aggregate data nor individual participants’ data were easily captured by a simple function (Figure 3) that we felt would explain the variance across morph levels. Second, we wished to examine response latency to correct categorization responses only, but in the middle of the morph continuum, there is not an objective correct answer. Therefore, we chose to use only the raw response latencies at each end point of the morph continuum and examined the impact of face category (real vs. doll) and orientation (upright vs. inverted) using a 2 × 2 repeated-measures analysis of variance (ANOVA).

This analysis revealed a significant main effect of face orientation, *F*(1, 23) = 12.66, *p* = .002, partial η^2 ^= 0.36, such that response latencies to upright faces were shorter than latencies to inverted faces (95% CI of the difference between means [0.018 s, 0.067 s]). Neither the main effect of category, *F*(1, 23) = 0.70, *p* = .41, partial η^2 ^= 0.03, nor the interaction between the two factors, *F*(1, 23) = 2.28, *p* = .14, partial η^2 ^= 0.09, reached significance.

### Discussion

Our results replicate some features of real versus artificial face categorization but also differ from previous reports in some ways. Like others (Balas & Horski, 2012; Looser & Wheatley, 2010), we observed a clear nonlinearity in the responses made across the morph continuum spanned by real and doll face appearance. Participants shift rapidly from labeling faces as being real to labeling them as doll faces near the midpoint of the animacy spectrum, which could reflect the presence of a category boundary in this region. Unlike prior reports, however, we did not observe that the position of this midpoint was significantly shifted toward the “Real” end of the continuum. This effect has been observed both with real/doll and real/CG morph continua and has been interpreted to mean that observers are conservative with their assignments of animacy: Only a little bit of artificial appearance is sufficient to reject a face as appearing real. This is consistent with “attractor field” models of categorization (Tanaka, Giles, Kremen, & Simon, 1998) in which an underrepresented category draws nearby stimuli to be categorized with that label by virtue of the large region of unoccupied face space around exemplars belonging to that group. The lack of this bias in our data may reflect a number of factors, particularly the brief presentation time that we used relative to prior studies. To our knowledge, the microgenesis of animacy categorization has not been characterized and so it may be the case that observers require additional time to extract sufficient information about face appearance to enforce the conservative labeling that leads to this shift in the psychometric function.

With regard to the impact of face inversion on categorization behavior, our data indicate that neither the steepness nor the midpoint of the underlying psychometric curve are affected by planar rotation. Instead, we observed only effects of inversion on the gamma and lambda parameters, which reflect behavior near the end points of the spectrum more than the midpoint (Klein, 2001). We will discuss the implications of this outcome more thoroughly in the general discussion, but for now simply point out that this indicates that the behavioral evidence for a steep boundary between real and artificial faces is equally apparent in upright and inverted images. For now, we continue by examining how inversion affects the neural processing of real and artificial faces by determining how this manipulation affects the response properties of the P100 and N170, two ERP components that are known to be sensitive to face appearance (Bentin, Allison, Puce, Perez, & McCarthy, 1996; Dering, Martin, Moro, Pegna, & Thierry, 2011).

## Experiment 2

In our second experiment, we examined the impact of face inversion on neural responses to real and artificial faces. Specifically, we measured the response properties of the P100 and N170 components (amplitude and latency) subject to the presentation of upright or inverted faces depicting real people or dolls. Both of these components are known to be sensitive to face orientation, and to some extent they both exhibit some sensitivity to face animacy. Because of these prior results, both responses are useful targets for examining the potential neural sensitivity of real/artificial face categorization to inversion. Specifically, does inverting face images affect the extent to which differential responses to real and artificial faces are observed? This experiment provides us with the opportunity to examine neural responses that likely reflect distinct stages of face processing, potentially allowing us to see an impact of inversion on the response to real and artificial faces that is difficult to see in behavioral tasks.

### Methods

#### Participants

Our final sample was comprised of 15 adult participants (8 female). All participants reported normal or corrected-to-normal vision and were strongly right-handed as assessed using the Edinburgh Handedness Inventory (Oldfield, 1971). As in Experiment 1, written informed consent was obtained from all participants prior to the beginning of the experimental session. All participants were naïve to the purpose of the experiment and had not taken part in Experiment 1.

#### Stimuli

We used a subset of the images described in Experiment 1 for this task. Specifically, we used only the images from the “end points” of the morph continua described previously, so that participants would see only entirely real or entirely artificial faces. The full stimulus set was thus comprised of eight images, each of which was depicted in both upright and inverted orientations. As in Experiment 1, these images were matched for mean pixel intensity. Also, inversion was carried out by rotating each face about a central point, meaning that upper and lower visual fields were approximately matched in terms of the amount of visual input.

#### Procedure

We recorded continuous EEG using a 64-Channel Hydrocel Geodesic Sensor Net (EGI) and an EGI NetAmps 200 amplifier. Each participant was fitted with an appropriately sized sensor net for EEG recording, which was soaked in KCl solution to facilitate measurements of electric potential at the scalp. Stable impedances below a threshold of 25 kΩ were established prior to the beginning of the recording session, and impedance was monitored throughout the session. EEG measurements were sampled at 250 Hz and referenced online to the vertex electrode (Cz).

Participants completed the recording session seated at a comfortable viewing distance (∼50 cm) from a 1024 × 768 LCD monitor. On each trial, participants were presented with a single image depicting either a real face or a doll face against a medium gray background, which could be presented upright or inverted. The order of trials was pseudorandomized for each participant. Each image subtended approximately 5 to 6 degrees of visual angle and was presented onscreen for 500 ms before disappearing. Participants were asked to indicate the category of each face (real or doll) using a button box held with both hands in their lap. The orientation of this box was flipped in half of our participants so that the correct button mappings for each trial would be matched across participants. The intertrial interval was randomly determined on each trial by sampling from a uniform distribution bounded by 500 ms to 1,000 ms. Each face image was presented 10 times in each orientation, for a grand total of 160 trials in the entire session. Participants typically completed the entire session in approximately 45 minutes.

### Results

All preprocessing steps were carried out using NetStation v4.0. To obtain average ERPs for each condition, we first applied a low-pass filter with a 30 Hz cut-off frequency to the continuous EEG data recorded during the entire session. Following this, we segmented individual trials using stimulus onset triggers, including a 100 ms prestimulus baseline period and a 900 ms postonset epoch. Each of these segments was then baseline corrected by subtracting the mean voltage measured during the prestimulus baseline period from the entire waveform. Next, we applied routines for ocular artifact detection and bad channel replacement. Finally, we obtained average ERPs within each stimulus category and rereferenced these to an average rereference computed across the entire sensor array.

**Figure 4.**
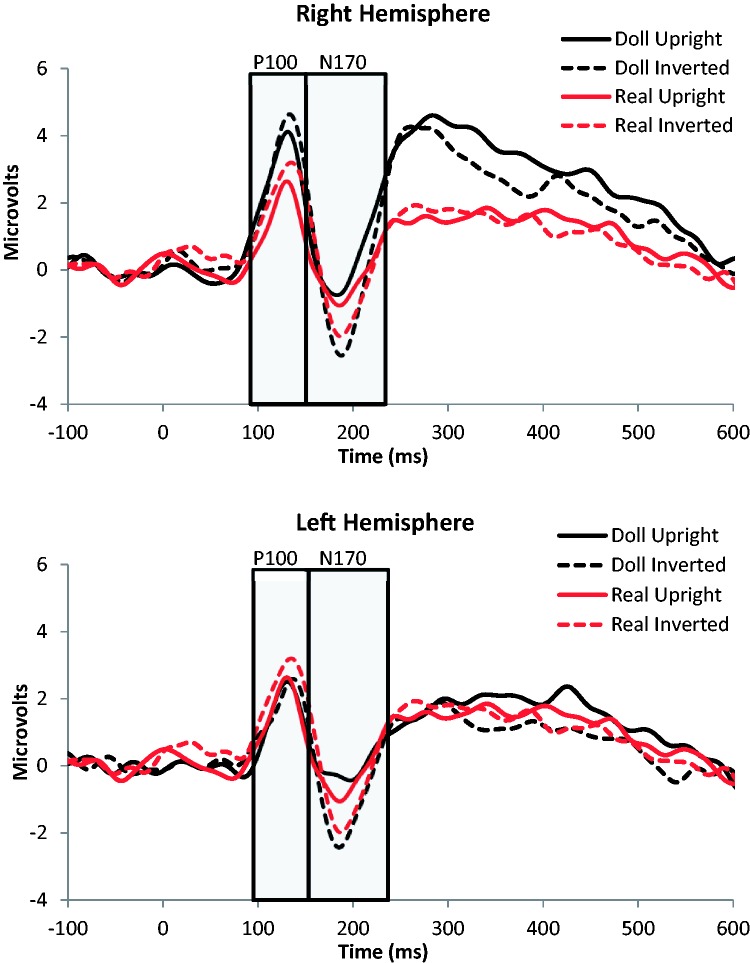
Grand average ERPs from the right hemisphere depicting both the P100 and N170 component for upright and inverted real and doll faces. ERP = event-related potential.

We identified sensors of interest for the N170 component by inspecting the grand average ERP collapsed across all participants and all conditions. Based on this visual analysis, we selected electrodes T5 and T6 in the left and right hemispheres as well as two adjacent electrodes in each hemisphere (LH: Electrodes 29, 30, and 32; RH: Electrodes 43, 44, and 47. These numbers correspond to the numbering scheme used for EGI’s 64-Channel Hydrocel Nets). Using these sensors, we obtained average left hemisphere and right hemisphere ERPs (Figure 4) for each condition by averaging together the waveforms observed within each group for each participant. We also identified time windows that captured variability across participants in the P100 (99 ms–159 ms) and N170 components (159 ms–223 ms), within which we calculated both the mean amplitude and peak latency. The latter was defined in terms of the time point at which the maximum amplitude occurred within the time window defined for each component. For each dependent variable, we used a 2 × 2 × 2 repeated-measures ANOVA with stimulus category (real vs. doll), orientation (upright vs. inverted), and hemisphere (left vs. right) as within-subjects factors. In the following, we report the outcome of these analyses for each component. Briefly, orientation was the only factor that significantly affected any measure, impacting the N170 amplitude and both the P100 amplitude and latency.

### N170

#### Amplitude

We observed only a main effect of stimulus orientation on the N170 amplitude, such that amplitude was greater (more negative) for inverted (*M* = −0.60, *SE* = 0.69) than upright (*M* = 0.52, *SE* = 0.73) stimuli (see Table 1, A for ANOVA results). Neither the main effects of stimulus category and hemisphere nor the interactions between these factors reached significance.

**Table 1.** Experiment 2 Results.

	Amplitude				Latency			
Factor	*df*	*F*	*p*	$\eta_{\text{p}}^{2}$	*df*	*F*	*p*	$\eta_{\text{p}}^{2}$
A. N170 results								
Animacy	1,14	0.10	.76	.01	1,14	0.74	.40	.05
Orientation	1,14	40.81	**<.001**	.75	1,14	0.83	.38	.86
Hemisphere	1,14	0.08	.79	.01	1,14	0.31	.59	.02
Animacy × Orientation	1,14	0.63	.44	.04	1,14	0.004	.95	.00
Animacy × Hemisphere	1,14	0.04	.84	.003	1,14	0.30	.60	.02
Orientation × Hemisphere	1,14	0.83	.38	.06	1,14	0.29	.60	.02
Animacy × Orientation × Hemisphere	1,14	2.51	.14	.15	1,14	1.53	.24	.10
B. P100 results								
Animacy	1,14	0.01	.91	.001	1,14	0.20	.66	.01
Orientation	1,14	11.55	**.004**	.45	1,14	9.34	**.01**	.40
Hemisphere	1,14	4.08	.06	.23	1,14	0.15	.71	.01
Animacy × Orientation	1,14	1.85	.20	.12	1,14	1.06	.32	.07
Animacy × Hemisphere	1,14	3.18	.10	.19	1,14	0.04	.85	.003
Orientation × Hemisphere	1,14	0.07	.79	.01	1,14	0.37	.55	.03
Animacy × Orientation × Hemisphere	1,14	0.08	.78	.01	1,14	0.69	.42	.05

Bold values represent *p* < 0.01 level.

#### Latency

None of our factors affected N170 latency, as the ANOVA revealed all null effects.

### P100

#### Amplitude

Again, we observed a main effect of stimulus orientation on the P100 amplitude, such that amplitude was greater (more positive) for the inverted (*M* = 3.42, *SE* = 0.53) than upright (*M* = 2.70, *SE* = 0.52) stimuli. We also observed a marginally significant effect of hemisphere on P100 amplitude, as amplitude tended to be higher in the right (*M* = 3.51, *SE* = 0.65) hemisphere than the left hemisphere (*M* = 2.61, *SE* = 0.46). No other main effects or interactions reached significance.

#### Latency

Finally, P100 latency was also affected only by orientation. We observed significantly slower latencies in response to inverted (*M* = 133.57, *SE* = 1.99) relative to upright (*M* = 129.23, *SE* = 2.50) stimuli.

## General Discussion

Across two experiments, we have demonstrated that face inversion has a fairly limited impact on categorization, considered both in terms of behavioral responses and neural indices of face processing. Behaviorally, our results do not suggest that the nature of the category boundary between real and artificial boundaries is shifted or attenuated by face inversion. Neither the point of subjective equality nor the steepness of the psychometric curve was significantly affected by turning morphed real/doll faces upside down. We did observe, however, a difference in effects at the real and doll ends of the spectrum (reflected in the gamma and lambda parameters obtained from our curve fits), indicating that inversion might affect real faces more than artificial faces. Participants’ response latencies did not reveal such an interaction between face category and inversion, but we do note that the difference between upright and inverted response times trended larger at the real end of the spectrum as well. Together, these features of the data provide some tentative reasons to conjecture that the inversion effect may be reduced for artificial faces relative to real faces. Were this the case (and we emphasize that our results are at best only suggestive of such an outcome), it would be an interesting extension of the aforementioned work demonstrating that other aspects of face expertise are not evident with artificial faces (Balas & Pacella, 2015; Crookes et al., 2015). More generally, expertise appears to affect face perception such that deprivation (Le Grand, Mondloch, Maurer, & Brent, 2004) or membership in an out-group face defined by race or age (Wiese, 2012) do not always elicit behavioral markers of expert-level processing, such as evidence of holistic processing (Michel, Rossion, Han, Chung, & Caldara, 2006) via the composite face effect (Richler, Floyd, & Gauthier, 2014; Young, Hellawell, & Hay, 1987) or the inversion effect (Balas & Nelson, 2010). Other categories, like species, that offer more profound differences in appearance across groups have been found to support such “special” features of face processing, however (Taubert, 2009), which offers interesting complications to any attempt to synthesize the literature describing how experience and exposure affect these aspects of face processing. Still, examining the inversion effect’s strength in real and artificial cases when face identification or other subordinate tasks are required would be an interesting direction for further study. Our results suggest that the process of categorizing faces as real or doll-like is not affected drastically by inversion, but other recognition tasks could certainly yield different outcomes.

Our ERP results further suggest that the categorization of faces as real versus artificial is not impacted much by face inversion. We observed robust FIEs in both appearance conditions, and no evidence of an interaction between animacy and face orientation at the N170 or P100. Indeed, we also observed no main effects of animacy at either component, which is consistent with prior ERP studies, suggesting that the distinction between real and artificial face appearance is primarily evident at later stages of processing (∼400 ms poststimulus onset; Looser et al., 2013). Thus, while animacy can disrupt neural sensitivity to different face categories defined by own- and other-species (Balas & Koldewyn, 2013), it appears that it does not disrupt the differential response to upright and inverted faces, nor does the inversion effect modulate how animacy is processed. In selecting the P100 and N170 in particular, we acknowledge that we are limiting our focus to relatively early stages of visual processing that are known to be face-sensitive and tacitly ignoring later stages of face recognition that may contribute. Given the vast literature describing the effects of inversion at these components, we think that this was a reasonable approach, but we also acknowledge that examining this issue (and even this data set) more broadly would be useful. In particular, applying techniques for multivariate pattern analysis to ERP data (Blankertz, Lemm, Treder, Haufe, & Muller, 2011) or other methods for modeling single-trial responses (Rousselet, Husk, Bennett, & Sekuler, 2007) could be a principled way to determine when sensitivity to animacy is affected by inversion (if at all) without the need to identify components at each candidate stage of processing. Presently, however, our data suggest that at least in terms of these markers of processing within the “face network” (Haxby, Hoffman, & Gobbini, 2000; Pitcher, Walsh, Yovel, & Duchaine, 2007), face inversion does not affect how real and artificial faces are categorized.

What do these outcomes imply about the nature of categorical processing for real and artificial faces? Our behavioral data in particular indicate that some caution regarding the evidence for true categorical processing along the real/doll continuum would be wise. If the steepness (or nonlinearity) of the psychometric function relating presumably linear changes in appearance to participant responses is a key piece of supporting data for categorical processing (Looser & Wheatley, 2010), the lack of an inversion effect on this curve is troubling. A steep boundary like this could be evidence of categorical processing but could also reflect nonlinearities along the morph continuum used to measure categorization behavior, which hopefully inversion would offer a useful control for. Our results demonstrating that the steepness of the boundary does not change with inversion are consistent with the possibility that low-level features may indeed differ more profoundly toward the middle of our morph continuum, leading to larger changes in behavior in this region as a result of image-level information rather than true face processing. A counter-argument to this account of the data is that inversion does not affect face processing qualitatively but only reduces the efficiency of face mechanisms for recognition (Sekuler, Gaspar, Gold, & Bennett, 2004). Thus, inverted faces may still be processed in the same manner as upright faces, meaning that the similar steepness of the psychometric curves we measured in both conditions might still reflect properties of face processing proper. To our thinking, neither explanation is sufficiently established in its own right for us to abandon the question, so we take our data as an indication that more detailed examination of how low-level image differences affect categorical processing for real versus artificial faces is necessary. In particular, we suggest that explicitly matching low-level features using tools like the SHINE toolbox (Willenbockel et al., 2010) is an important means of controlling for the possibility that the morph continuum itself may have nonlinear changes built into it. Also, measuring ostensibly categorical behavior using both explicit categorization judgments like we have employed here and discrimination behavior for images that lie on the same side of the category boundary versus images that lie on opposite sides of it (Beale & Keil, 1995) would provide useful complementary information about the nature of this candidate boundary. Finally, another interesting way to interpret our results is to conjecture that the lack of an inversion effect for real/artificial categorization may indicate that this task does not rely on holistic processing. The inversion effect itself has been interpreted by some as the result of a failure to apply holistic processing to inverted faces, which further implies that inverted faces are processed by part-based mechanisms. To the extent that one accepts this framework, our data could suggest that categorizing faces as real or artificial relies only on part-based mechanisms and does not require holistic processing at all. Examining how animacy categorization is impacted by the presentation of face parts in isolation could be a useful way to further explore this idea and establish what basic processes contribute to the ability to determine if a face is real or not.

Overall, these results have important implications for our understanding of categorical processing of face animacy. Our data highlight the need for a more thorough examination of the evidence suggesting that artificial faces are truly a distinct category of faces but also offer some preliminary evidence supporting the idea that artificial faces may not just be a distinct category of faces but also an “out-group.” This latter issue has important consequences for the many studies that have made heavy use of various kinds of artificial faces to examine aspects of face perception including, development, face identification, and social cognition (Oosterhof & Todorov, 2008). Continued study of the relationship between the processing of real faces and artificial faces of different types thus merits attention and has the potential to address important theoretical issues and speak to practical concerns regarding the methodological use of synthetic faces of multiple types.
